# Fracture of Lithia Disilicate Ceramics under Different Environmental Conditions

**DOI:** 10.3390/ma15155261

**Published:** 2022-07-29

**Authors:** Josephine F. Esquivel-Upshaw, Shu-Min Hsu, Fan Ren, Jenna Stephany, Xinyi Xia, Chan-Wen Chiu, Dan Neal, John J. Mecholsky

**Affiliations:** 1 Division of Prosthodontics, College of Dentistry, Restorative Dental Sciences, University of Florida, Gainesville, FL 32610, USA; dai760705@gmail.com (S.-M.H.); jstephany@dental.ufl.edu (J.S.); 2 Department of Chemical Engineering, Herbert Wertheim College of Engineering, University of Florida, Gainesville, FL 32610, USA; fren@che.ufl.edu (F.R.); xiaxinyi@ufl.edu (X.X.); chanwen.chiu@ufl.edu (C.-W.C.); 3 Department of Surgery, College of Medicine, University of Florida, Gainesville, FL 32610, USA; dneal@ufl.edu; 4 Department of Materials Science and Engineering, Herbert Wertheim College of Engineering, University of Florida, Gainesville, FL 32610, USA; jmech@mse.ufl.edu

**Keywords:** fracture strength, ceramic corrosion, surface degradation

## Abstract

The objective of this research was to quantify the effect of surface degradation and abrasion separately and in combination on the flexural strength of lithia disilicate ceramics. Lithia disilicate disks were fabricated using the lost wax technique and pressing in vacuum. The eight groups in this pilot experiment were (i) reference, hydrated in distilled water for 24 h prior to fracture; (ii) reference, non-hydrated group; (iii) 28-day pH cycling group; (iv) 125K chewing cycle group; (v) combined pH cycling + 125K chewing cycle; (vi) constant pH 2 solution for 28 days; (vii) constant pH 7 solution for 28 days; and (viii) constant pH 10 solution for 28 days. pH cycling is a method that alternates between pH 2, 7 and 10 over 28 days. A total of 15 disks were used for each group. All the groups were tested using the biaxial piston and a three-ball flexural strength test to obtain their biaxial flexural strength. pH 2 constant immersion demonstrated the highest fracture strength and was significantly greater than all other groups (*p* < 0.0001). Chewing and pH cycling + chewing groups exhibited the lowest fracture strengths and were significantly lower than all other groups (*p* < 0.0001). The damage observed from the chewing simulator does not represent apparent clinical fractures.

## 1. Introduction

Fracture resistance and chemical durability are two of the most important requirements for dental restorations. With the constant forces of mastication from opposing enamel, coupled with changes in pH as a result of diet and saliva, dental restorations are expected to withstand harsh and constantly changing environments. ISO 6872, which is the standard for ceramic materials, measures the chemical durability through exposure to pH 2 for several hours. However, foods ingested have pH levels ranging from acidic to basic. An in vitro study demonstrated that pH 10 and pH 7 are more detrimental to the surface integrity of ceramics than pH 2 [1]. In addition, a method of pH cycling, where pH 2, 7 and 10 were applied alternately over a period of time, was more detrimental to the ceramic surface than applying these solutions constantly [2]. Ceramic corrosion has been shown to adversely affect the fracture resistance of these materials [3,4] and their surface integrity [5].

All-ceramic prostheses have a reported success rate that is significantly lower than that of metal-ceramic prostheses [6,7,8,9]. Technical complications refer typically to veneer chipping and core fracture of these all-ceramic prostheses [10,11,12]. We conducted a randomized clinical study that identified design parameters that might affect the survival of veneered implant-supported all-ceramic and metal-ceramic fixed dental prostheses (FDPs) [13]. The results of this study revealed that after five years of service, 16% of these implant-supported prostheses failed. Of these failures, 35% were metal-ceramic control FDPs and 65% were all-ceramic FDPs. The main cause of failure was attributed to chipping fractures, which occurred primarily on the occlusal surface of the prostheses, limited to the veneer [14], and were associated with maximum intercuspation contacts (*p* = 0.004). Factors such as veneer thickness, connector height and radius of curvature of the gingival embrasure were not associated with the incidence of fractures. However, maximum wear was observed on the occlusal surface of the ceramic FDPs, averaging 42.1 ± 31.9 µm (6 mos) to 85.0 ± 48.4 µm (1 yr) intraorally [15], indicating substantial degradation of the ceramic surface.

The objective of this research was to determine the effect of cycling pH and chewing, individually and combined, on the fracture strength of lithia disilicate ceramics. The long-term objective is to develop in vitro testing methodologies that will simulate the conditions in the oral cavity.

## 2. Materials and Methods

### 2.1. Sample Preparation

All the samples (12 mm × 1.5 mm) were fabricated according to the manufacturer’s instructions for pressing ceramics. Disks were made using the lost wax technique, invested and pressed (Emax Press, Ivoclar Vivadent, Schaan, Liechtenstein). The invested mold was further sandblasted with 80 μm glass beads (Williams’ glass beads, Ivoclar Vivadent, Schaan, Liechtenstein) at 58 psi pressure to retrieve the glass-ceramic disks with sprues. A diamond-cutting saw was used to cut the sprues. The pressing disks were immersed in Invex liquid and polished using 320, 400, and 600 grits successively for both sides (Ecomet 250, Buehler, Lake Bluff, IL, USA). After polishing, the disks were cleaned in the ultrasonic cleaner using alcohol and D.I. water.

### 2.2. Experimental Design

The eight groups in this pilot experiment were (i) reference, hydrated in distilled water for 24 h prior to fracture; (ii) reference, non-hydrated group; (iii) 28-day pH cycling group; (iv) 125K chewing cycle group; (v) combined pH cycling + 125K chewing cycle; (vi) constant pH 2 solution for 28 days; (vii) constant pH 7 solution for 28 days; and (viii) constant pH 10 solution for 28 days. The pH corrosion and chewing tests will be introduced in the following sections in detail (Section 2.3 and Section 2.4). There are 15 disks for each group. All the groups were tested using the biaxial piston and three-ball flexural strength test (Section 2.5) to obtain their biaxial flexural strength [16].

### 2.3. pH Corrosion Test

The corrosion tests were performed in either constant immersion or pH cycling conditions for 28 days. pH 10 (potassium carbonate-potassium borate-potassium hydroxide buffer, SB116-500, Fisher Chemical, Pittsburgh, PA, USA), pH 2 (Potassium Chloride-Hydrochloric acid, SB96-500, Fisher Chemical, Pittsburgh, PA, USA), and pH 7 (Potassium Phosphate Monobasic-Sodium Hydroxide, SB108-500, Fisher Chemical, Pittsburgh, PA, USA) buffer solutions were used in this study. Constant immersion involved leaving the disks immersed in either pH 2, pH 7 or pH 10 buffer solutions continuously for 28 days. pH cycling involved alternating between the different buffer solutions. The disks for pH cycling were immersed using the sequence pH 10, pH 7, pH 2 and pH 7, pH 10 and so forth, where the solutions were changed every day. All the disks were placed in polyethylene centrifuge tubes (Thermo Scientific Nalgene Oak Ridge High-Speed Centrifuge Tubes, Thermo Fisher Scientific, Waltham, MA, USA) and 80 °C using the shaking water bath (water bath shaking TSBS40, Techne, Minneapolis, MN, USA) with 50 oscillations per minute.

### 2.4. Chewing Simulation Test

The wear was performed using a commercial chewing simulator (CS-4, SD Mechatronik, Pleidelsheim, Germany). There are eight chambers with load cells. The disks were mounted on the customized holders and were fastened in the chambers. The antagonistic balls were embedded in the upper holders. Masticatory force was simulated by controlling the horizontal and vertical movements of disks and antagonistic balls. In total, 125,000 chewing cycles were performed with 49 N loading force. The parameters are listed in Table 1. The antagonistic balls move downward to the disks using the upper crosshead. When the antagonistic balls touch the disks, the loading force was transferred to each disk individually. The disks were then moved horizontally, which allows antagonist balls to be moved back to the previous position to complete the mastication cycle. The testing chamber was filled with D.I. water and cycled at 25 °C.

### 2.5. Biaxial Flexural Strength

The biaxial flexural strengths were determined on a universal testing machine with a crosshead speed rate of 1 mm/min using a piston on a 3-ball apparatus in room temperature conditions. A total of 15 disks were used for each group. After alignment, the disks were placed on 3 symmetrical balls (2 mm in diameter). A thin PTFE film (0.05 mm thick) was applied between the piston (diameter) and the disks to distribute the contact pressure. Adhesive tape was placed between the disks and the balls to prevent lateral movement. The maximum biaxial flexure strength was calculated using Equation (1) [16]:

(1) $$\sigma_{f}=-0.2387\;P\;\left(X-Y\right)/d^{2}$$

where $\sigma_{f}$ is the maximum tensile stress at center (MPa); **P** is the load at fracture (N); ***d*** is the disk’s thickness at fracture origin (mm); **X** and **Y** are described in Equations (2) and (3):

(2) X=(1+ν)Ln(r2r3)2+[(1−ν)2](r2r3)2

(3) Y=(1+ν)[1+Ln(r1r3)2]+(1−ν)(r1r3)2

where **ν** is Poisson’s ratio; $r_{1}$ is the radius of the support of the 3 balls (mm); $r_{2}$ is the radius of the piston’s loaded area (mm); $r_{3}$ is the radius of the disks (mm). The strengths were analyzed using Weibull statistics [17]. In that analysis, the Ln Ln ((1/(1 − Failure Probability)) is graphed against Ln (strength). The slope of the graph indicates the dispersion of the data and is called the Weibull modulus. Low numbers indicate a wider dispersion than greater numbers.

### 2.6. SEM Analysis

Scanning Electron Microscopy (Hitachi S-3000, Hitachi High-Tech America, Inc., Schaumburg, IL, USA) was performed at 2 KV with a working distance of 3.9 mm on all fractured surfaces which were carbon coated to determine the quality of the interface after treatments as well as to determine the origins of fracture for the disks. The magnification varied from 100× to 500×. Scale bars were added to indicate scale.

### 2.7. Statistical Analysis

For each test group, strength was summarized as median (i.e., 25th percentile, 75th percentile) (range). Mann–Whitney tests (non-parametric *t*-tests) were used to make pairwise comparisons between groups. All analyses were performed using the R statistical software package (V.4.0.2) (Vienna, Austria).

## 3. Results

**Figure 1:**
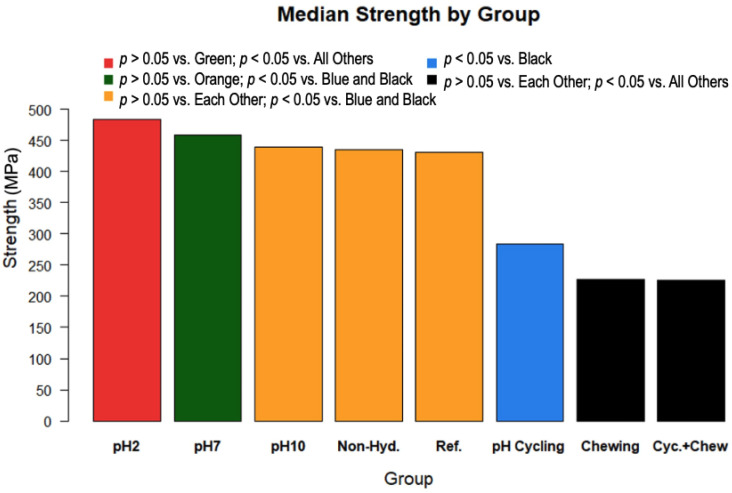
Fracture strength data in MPa, comparing and ranking strengths by test group.

The results of the strength tests are shown in Figure 1. These results demonstrate that pH 2 had the highest fracture strength and was significantly greater than all groups with the exception of pH 7 (*p* < 0.0001). pH 7 had the second to highest fracture strength and pH 10 had the third highest. Both were significantly greater than pH cycling, pH cycling + chewing and chewing-only groups (*p* < 0.0001). Chewing-only and pH cycling + chewing groups had almost identical strength values and were significantly lower than all other groups (*p* < 0.0001).

**Figure 2:**
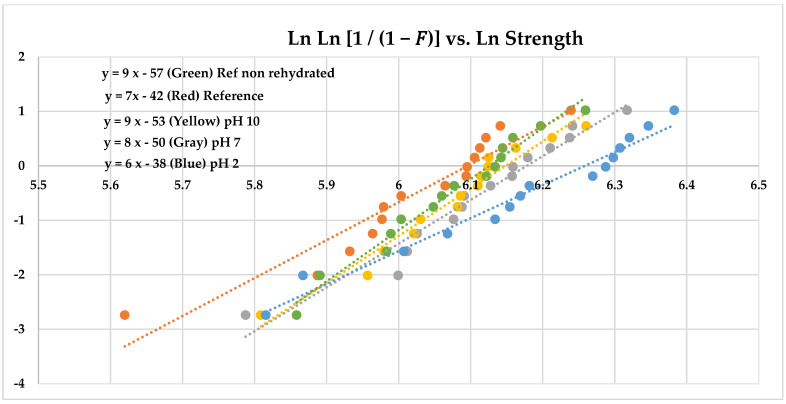
Weibull distribution showing pH 2 with a greater strength distribution.

**Figure 3:**
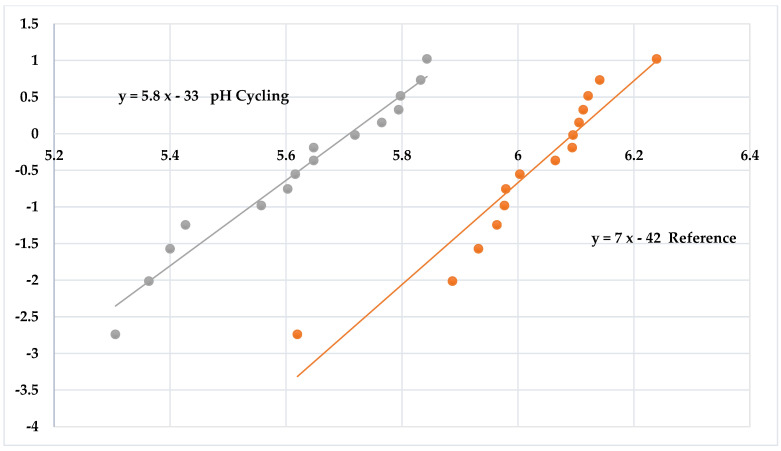
Weibull distribution comparison between pH cycling and reference (hydrated) groups (Ln Ln [1/(1 − F)] vs. Ln Strength).

The Weibull graphs for the reference disks (hydrated) and pH treatments are shown in Figure 2. The pH 2 data separate from the other data, indicating a greater strength distribution. The comparison of the pH cycling to the reference disks as Weibull graphs is shown in Figure 3. These further illustrate the significant difference between the two groups, with pH cycling treatment causing significantly decreased strength.

**Figure 4:**
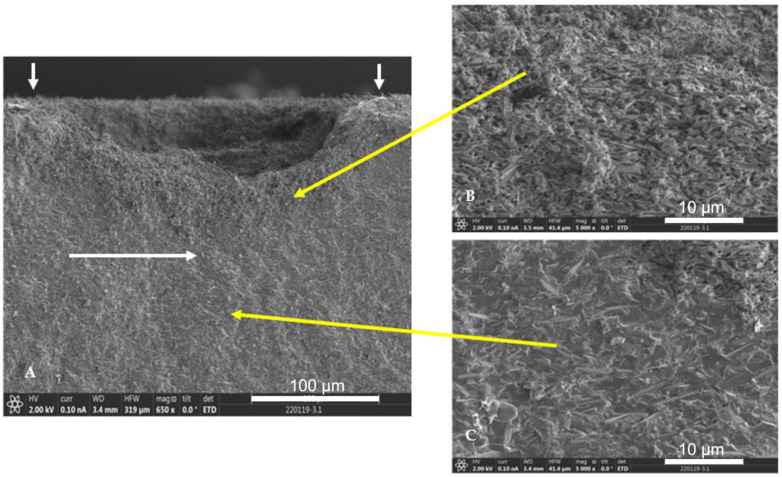
(**A**) SEM image of area around fracture origin for the pH cycling group. White arrows demarcate extent of corrosion layer; (**B**) higher magnification of alteration layer with dissolution of glassy phase of the material; (**C**) higher magnification of area below the alteration layer with crystals and glassy phase intact.

SEM analysis of fractured interface for pH cycled disks shows a corrosion layer (Figure 4), which was not evident on the other disks with constant immersion.

**Figure 5:**
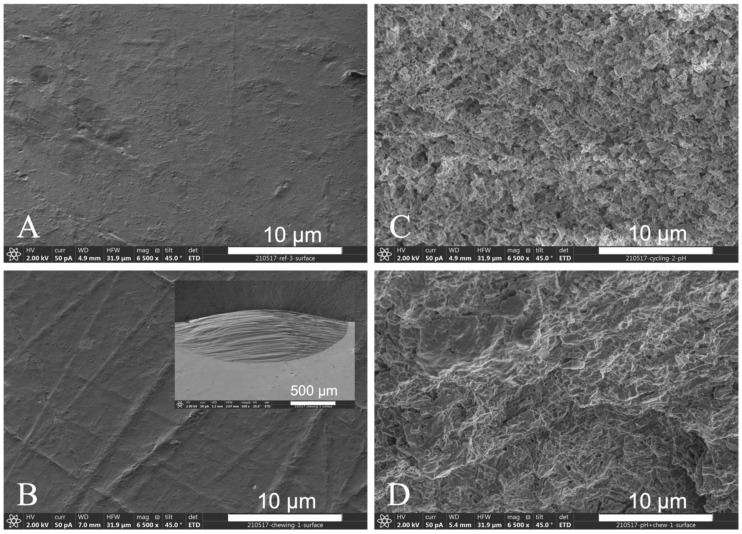
SEM analysis of surfaces of (**A**) reference; (**B**) chewing only (gouge on surface shown on inset); (**C**) pH cycling; (**D**) pH cycling + chewing. Line in (**A**) is 10 μm for (**B**–**D**). Bar in inset is 500 μm.

SEM analysis of the surface of disks after treatment demonstrated extensive surface degradation for pH cycled groups (Figure 5). Chewing groups have extensive gouging on the surface of the disks (Figure 5B).

## 4. Discussion

The oral environment presents a major challenge for ceramic dental prostheses because the pH ranges from extremely acidic to extremely basic conditions. There are currently no standardized tests that simulate these intra-oral changes in pH that can be influenced by diet and the buffering capacity of saliva. Studies have demonstrated the significant surface degradation that occurs when ceramics are immersed in pH 2, 7 and 10 independently [1]. Other studies demonstrated that the effects on the surface are more deleterious when pH 2, 7 and 10 are cycled, that is, when samples are immersed in alternating pH solutions [2,18]. The researchers proposed a mechanism that occurs in glass-ceramics when pH environments are alternated, which is not only limited to ionic exchange but extends to dissolution of the silicon bonds and cleavage of the whole material. The flexural strength of most ceramics has been shown to decrease when subjected to harsh environments while under stress [19,20], such as those seen in the oral cavity.

This study demonstrates that environmental conditions, namely pH levels, can affect the strength properties of dental ceramics. Previous studies have shown the effect of chemical environment on surface degradation of ceramic surfaces. However, there are limited data on the effect of these environments on fracture strength. Constant immersion in pH 2 demonstrated a less degraded surface compared to pH 7 and pH 10 [1]. However, in this same study, samples immersed in all pH levels exhibited more surface degradation than the reference samples. Based on this, one would expect the reference disks to exhibit a greater fracture resistance than all other disks subjected to pH changes. In this current study, the average strength of the pH 2 constant exposure samples statistically increased by about 10% compared to the reference values and were statistically greater than all the groups, with the exception of the pH 7 group (Figure 1). An explanation for the increased strength for pH 2 is that the solution blunted the sharp cracks that resulted from polishing the disks. Acid etching of cracks has been shown to round out the crack geometry and increase strength [21]. The other constant exposure strengths, i.e., pH 7 and pH 10, were the same as the reference strength. There is a difference between the reference disks and the pH cycling in that the pH cycling has less strength than the reference disks. Since the pH 2 data have greater strengths than the reference disks and the pH 7 and pH 10 constant immersion data have similar strengths to the reference disks, this implies that there is a synergistic effect in the combination of pH values that reduces the strength of the glass-ceramic disks. 

These glass ceramics in general are resistant to chemical environments. However, the strengths of the specimens exposed to pH cycling were about 50% less than the reference strengths. This agrees with earlier findings of the effect of pH cycling versus constant exposure on chemical durability [2,18]. These studies demonstrated that the increased frequency of pH changes significantly enhanced the corrosion of the glass-ceramic specimens. The alteration layer typically produced during an ionic exchange mechanism significantly affected the corrosion processes by hindering the release of ions into solution. The methodology of cycling pH with consistent pH changes disrupted the formation of an alteration layer and resulted in more weight loss and ion release during these cycling conditions (Figure 4). The effect of pH cycling on the strength has not been reported previously. Analyzing this phenomenon is critical to the understanding of the clinical behavior of dental protheses. Establishing the stress corrosion parameters for the condition of pH cycling is critical to designing an effective proof testing procedure.

The chewing and pH cycling + chewing samples all demonstrated large gouges on the surface of the glass ceramics (Figure 5). This is much more severe than any observed damage on crowns or bridges. A previous study performed forensic analysis on clinically fractured bridges through fractography, to determine the origin and cause of failure [14,22,23]. Clinical fractures were thought to have occurred as a result of repeated crack growth due to initial overload and continuous use after initial cracking. None of the clinical fractures resembled areas of gouging on the surface of the restorations. For this current study, the actual origins of the fracture were within the gouges produced by the chewing simulator on the surface. Thus, in our opinion, the damage from the chewing simulator does not mimic the damage from mastication that can lead to fracture. The results are outliers from the expected behavior and should not be statistically grouped with the other data. This explains the reason for both the chewing and pH cycling + chewing groups having identical fracture strengths. The cracks that cause failure in the chewing condition are much greater than any effect the other environmental conditions may have, such as pH cycling. A more subtle test of the effect of pH cycling must be developed. The chewing simulator is too aggressive and the cracks produced are much greater than normally observed.

## 5. Conclusions

There was a greater strength distribution for pH 2 than for pH 7 and pH 10 treatments with 28-day exposure compared to the reference material.

Chewing simulation resulted in lower strengths than the reference strengths due to large gouges in the material with random fracture origins that were different from those found in the pH solutions and reference material.

There was no statistical difference in strengths for chewing and pH cycling plus chewing, indicating that the effect of the damage from the chewing simulator masked any potential effect of the pH cycling.

The pH cycling resulted in lower strengths compared to the reference values as well as the pH (28 days) solutions. More testing of the pH cycling should be performed in order to understand how this combination of pH cycling affects strengths of dental glass ceramics.

## Figures and Tables

**Table 1:** Testing parameters for chewing simulation.

Vertical Ascending Speed (mm/s)	60
Vertical descending speed (mm/s)	60
Vertical ascending movement (mm)	2
Vertical descending movement (mm)	1
Horizontal speed (mm/s)	40
Horizontal movement (mm)	0.7
Loading force per sample (N)	49
Cycle frequency (Hz)	1.5

## Data Availability

Data available upon request.
